# Supplementary material for: Relationships between Maternal Gene Polymorphisms in One Carbon Metabolism and Adverse Pregnancy Outcomes: A Prospective Mother and Child Cohort Study in China
Source: Nutrients. 2022 May 18;14(10):2108. doi: 10.3390/nu14102108 (PMC9146434; doi:10.3390/nu14102108)
Supplement: Supplementary file 1 [file nutrients-14-02108-s001.zip › nutrients-1670789-supplementary.pdf]

Table S1. Sensitive analysis-Relationship between genotypes of MTHFR、 MTRR、MTR、TYMS and PTB in women whose SNPs genotyped description of “A. Conservative”.

Genotype		Controls [n(%)]	PTB [n(%)]	OR (95% CI)	*P*
MTHFR C677T	CC	84(96.55)	3(3.45)	1	-
	CT+TT	597(96.14)	24(3.86)	1.13(0.33- 3.82)	0.849
MTHFR A1298C	AA	543(96.11)	22(3.89)	1	-
	AC+CC	75(97.40)	2(2.60)	0.66(0.15- 2.86)	0.576
MTRR A66G	AA	396(95.65)	18(4.35)	1	-
	AG+GG	51(96.23)	2(3.77)	0.86(0.20- 3.83)	0.846
MTR A2756G	AA	561(96.72)	19(3.28)	1	-
	AG+GG	128(94.12)	8(5.88)	1.85(0.79- 4.31)	0.157
TYMS rs3819102	AA	355(96.73)	12(3.27)	1	-
	AG+GG	249(96.89)	8(3.11)	0.95(0.37- 2.36)	0.913

MTHFR, Methylenetetrahydrofolate reductase; *MTRR*, methionine synthase reductase; *MTR*, methionine synthase; *TYMS*, thymidylate synthetase; *PTB*, preterm birth

Table S2: Sensitive analysis-Relationship between genotypes of MTHFR、MTRR、MTR、TYMS and LBW in women whose SNPs genotyped description of "A. Conservative"

Genotype		Controls [n(%)]	LBW [n(%)]	OR (95% CI)	*P*
MTHFR C677T	CC	82(100.00)	0(0)	1	-
	CT+TT	594(98.18)	11(1.82)	-	0.973
MTHFR A1298C	AA	538(98.18)	10(1.82)	1	-
	AC+CC	75(100.00)	0(0)	-	0.960
MTRR A66G	AA	394(98.25)	7(1.75)	1	-
	AG+GG	52(100.00)	0(0)	-	0.968
MTR A2756G	AA	554(98.58)	8(1.42)	1	-
	AG+GG	130(97.74)	3(2.26)	1.60(0.42-6.11)	0.493
TYMS rs3819102	AA	345(97.73)	8(2.27)	1	-
	AG+GG	250(99.21)	2(0.79)	0.35(0.07- 1.64)	0.181

MTHFR, Methylenetetrahydrofolate reductase; *MTRR*, methionine synthase reductase; *MTR*, methionine synthase; *TYMS*, thymidylate synthetase; *LBW*, low birth weight

Table S3: Sensitive analysis-Relationship between genotypes of MTHFR、MTRR、MTR、TYMS and SGA in women whose SNPs genotyped description of “A. Conservative”

Genotype		Controls [n(%)]	SGA [n(%)]	OR (95% CI)	*P*
MTHFR C677T	CC	78(92.86)	6(7.14)	1	-
	CT+TT	572(94.86)	31(5.14)	0.71(0.29-1.74)	0.449
MTHFR A1298C	AA	519(94.54)	30(5.46)	1	-
	AC+CC	70(94.59)	4(5.41)	0.99(0.34- 2.89)	0.983
MTRR A66G	AA	379(93.81)	25(6.19)	1	-
	AG+GG	50(100.00)	0(0)	-	0.961
MTR A2756G	AA	534(94.85)	29(5.15)	1	-
	AG+GG	124(93.94)	8(6.06)	1.19(0.53- 2.66)	0.676
TYMS rs3819102	AA	334(93.56)	23(6.44)	1	-
	AG+GG	241(96.40)	9(3.60)	0.54(0.25-1.19)	0.128

MTHFR, Methylenetetrahydrofolate reductase; *MTRR*, methionine synthase reductase; *MTR*, methionine synthase; *TYMS*, thymidylate synthetase; *SGA*, small-for-gestational-age

Table S4: Sensitive analysis-Relationship between genotypes of MTHFR、 MTRR、MTR、TYMS and PTB in women whose delivery information only from delivery medical records

Genotype		Controls [n(%)]	PTB [n(%)]	OR (95% CI)	*P*
MTHFR C677T	CC	66(98.51)	1(1.49)	1	-
	CT+TT	463(98.93)	5(1.07)	0.71(0.08- 6.20)	0.759
MTHFR A1298C	AA	417(98.82)	5(1.18)	1	-
	AC+CC	112(99.12)	1(0.88)	0.75(0.09- 6.44)	0.789
MTRR A66G	AA	310(99.04)	3(0.96)	1	-
	AG+GG	219(98.65)	3(1.35)	1.42(0.28- 7.08)	0.672
MTR A2756G	AA	428(98.85)	5(1.15)	1	-
	AG+GG	101(99.02)	1(0.98)	0.85(0.10- 7.33)	0.881
TYMS rs3819102	AA	341(99.42)	2(0.58)	1	-
	AG+GG	188(97.92)	4(2.08)	3.63(0.66- 20.00)	0.139

MTHFR, Methylenetetrahydrofolate reductase; *MTRR*, methionine synthase reductase; *MTR*, methionine synthase; *TYMS*, thymidylate synthetase; *PTB*, preterm birth

Table S5: Sensitive analysis-Relationship between genotypes of MTHFR、MTRR、MTR、TYMS and LBW in women whose delivery information only from delivery medical records

Genotype		Controls [n(%)]	LBW [n(%)]	OR (95% CI)	*P*
MTHFR C677T	CC	67(100.00)	0(0)	1	-
	CT+TT	463(98.93)	5(1.07)	-	0.971
MTHFR A1298C	AA	417(98.82)	5(1.18)	1	-
	AC+CC	113(100.00)	0(0)	-	0.962
MTRR A66G	AA	311(99.36)	2(0.64)	1	-
	AG+GG	219(98.65)	3(1.35)	2.13(0.35- 12.86)	0.410
MTR A2756G	AA	429(99.08)	4(0.92)	1	-
	AG+GG	101(99.02)	1(0.98)	1.06(0.12-9.60)	0.957
TYMS rs3819102	AA	339(98.83)	4(1.17)	1	-
	AG+GG	191(99.48)	1(0.52)	0.44(0.05- 4.00)	0.469

MTHFR, Methylenetetrahydrofolate reductase; *MTRR*, methionine synthase reductase; *MTR*, methionine synthase; *TYMS*, thymidylate synthetase; *LBW*, low birth weight

Table S6: Sensitive analysis-Relationship between genotypes of MTHFR、MTRR、MTR、TYMS and SGA in women whose delivery information only from delivery medical records

Genotype		Controls [n(%)]	SGA [n(%)]	OR (95% CI)	*P*
MTHFR C677T	CC	65(97.01)	2(2.99)	1	-
	CT+TT	457(97.65)	11(2.35)	0.78(0.17-3.61	0.753
MTHFR A1298C	AA	411(97.39)	11(2.61)	1	-
	AC+CC	111(98.23)	2(1.77)	0.67(0.15-3.08)	0.610
MTRR A66G	AA	303(96.81)	10(3.19)	1	-
	AG+GG	219(98.65)	3(1.35)	0.42(0.11-1.53)	0.186
MTR A2756G	AA	424(97.92)	9(2.08)	1	-
	AG+GG	98(96.08)	4(3.92)	1.92(0.58- 6.37)	0.285
TYMS rs3819102	AA	332(96.79)	11(3.21)	1	-
	AG+GG	190(98.96)	2(1.04)	0.32(0.07-1.45)	0.139

MTHFR, Methylenetetrahydrofolate reductase; *MTRR*, methionine synthase reductase; *MTR*, methionine synthase; *TYMS*, thymidylate synthetase; *SGA*, small-for-gestational-age
